# {μ-*N*,*N*′-Bis[(*E*)-4-pyridylmethyl­idene]naphthalene-1,5-diamine}bis­[dichlorido(dimethyl sulfide)platinum(II)]

**DOI:** 10.1107/S1600536808024914

**Published:** 2008-08-09

**Authors:** Hyun Sue Huh, Soon W. Lee

**Affiliations:** a Department of Chemistry (BK21), Sungkyunkwan University, Natural Science Campus, Suwon 440-746, Republic of Korea

## Abstract

The title dinuclear platinum compound, [Pt_2_Cl_4_(C_22_H_16_N_4_)(C_2_H_6_S)_2_], with a long bridging bipyridyl-type ligand, is centrosymmetric and the Pt^II^ cation shows a slightly distorted square-planar coordination geometry. The Cl ligands are *trans* to each other, with a Cl—Pt—Cl angle of 178.83 (8)°. The pyridine ring forms a dihedral angle of 48.8 (2)° with the planar PtCl_2_SN unit. Within the mol­ecule, the distance between Pt atoms is 20.262 (5) Å and the N⋯N separation between the terminal pyridyl rings is 16.23 (1)Å.

## Related literature

For related literature, see: Barnett & Champness (2003 ▶); Costa *et al.* (2003 ▶); Han & Lee (2004 ▶); Hill *et al.* (1998 ▶); Huh *et al.* (2008 ▶) and references therein; Kinnunen *et al.* (2002 ▶); Leininger *et al.* (2000 ▶); Min *et al.* (2006 ▶); Kinnunen *et al.* (2002 ▶); Leininger *et al.* (2000 ▶); Min *et al.* (2006 ▶).
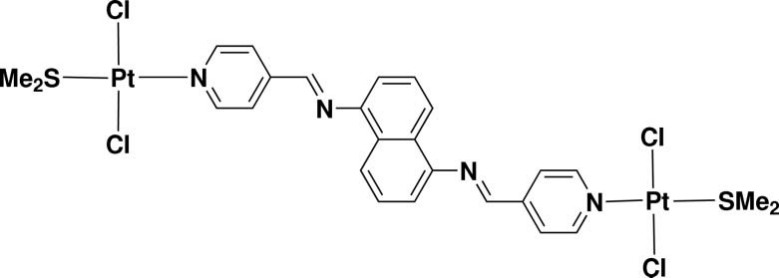


## Experimental

### 

#### Crystal data

[Pt_2_Cl_4_(C_22_H_16_N_4_)(C_2_H_6_S)_2_]
*M* *_r_* = 992.62
Triclinic, 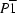

*a* = 5.172 (2) Å
*b* = 7.2482 (11) Å
*c* = 20.728 (3) Å
α = 91.596 (12)°
β = 91.974 (19)°
γ = 97.804 (17)°
*V* = 769.0 (3) Å^3^
*Z* = 1
Mo *K*α radiation
μ = 9.59 mm^−1^
*T* = 293 (2) K
0.44 × 0.20 × 0.10 mm

#### Data collection

Siemens P4 diffractometer
Absorption correction: ψ scan (North *et al.*, 1968 ▶) *T*_min_ = 0.115, *T*_max_ = 0.383
3026 measured reflections
2696 independent reflections
2447 reflections with *I* > 2σ(*I*)
*R*_int_ = 0.042
3 standard reflections every 97 reflections intensity decay: none

#### Refinement

*R*[*F* ^2^ > 2σ(*F* ^2^)] = 0.036
*wR*(*F* ^2^) = 0.093
*S* = 1.05
2696 reflections
172 parameters
H-atom parameters constrained
Δρ_max_ = 0.73 e Å^−3^
Δρ_min_ = −0.96 e Å^−3^

### 

Data collection: *XSCANS* (Siemens, 1995 ▶); cell refinement: *XSCANS*; data reduction: *SHELXTL* (Sheldrick, 2008 ▶); program(s) used to solve structure: *SHELXTL*; program(s) used to refine structure: *SHELXTL*; molecular graphics: *SHELXTL*; software used to prepare material for publication: *SHELXTL*.

## Supplementary Material

Crystal structure: contains datablocks global, I. DOI: 10.1107/S1600536808024914/gk2157sup1.cif

Structure factors: contains datablocks I. DOI: 10.1107/S1600536808024914/gk2157Isup2.hkl

Additional supplementary materials:  crystallographic information; 3D view; checkCIF report

## Figures and Tables

**Table:** 

Pt1—N1	2.057 (6)
Pt1—S1	2.285 (2)
Pt1—Cl2	2.301 (2)
Pt1—Cl1	2.303 (2)

**Table:** 

N1—Pt1—S1	175.98 (18)
N1—Pt1—Cl2	88.55 (19)
S1—Pt1—Cl2	95.31 (8)
N1—Pt1—Cl1	90.40 (19)
S1—Pt1—Cl1	85.74 (8)
Cl2—Pt1—Cl1	178.83 (8)

**Fig. 1.**
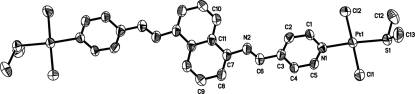
Molecular structure of the title compound showing 50% probability displacement ellipsoids. H atoms are omitted for clarity. Unlabeled atoms are related to labeled atoms by the symmetry operation i = -x + 1, -y + 1, -z + 1.

## supplementary crystallographic information

### Comment

The N-donor linking (*exo* N-donor bidentate) ligands such as pyrazine, 4,4'-bipyridine, 1,2-bis(4-pyridyl)ethylene, and 1,2-bis(4-pyridyl)ethane are widely used for the synthesis of discrete dinuclear, polynuclear, and coordination network compounds (Leininger *et al.*, 2000; Kinnunen *et al.*, 2002; Costa *et al.*, 2003; Barnett & Champness, 2003). We also reported dinuclear discrete rods, tetranuclear rectangles, and one-dimensional coordination networks of Cp*Rh(III) compounds by employing such ligands, where Cp* is 1,2,3,4,5-pentamethylcyclopentadiene (Han & Lee, 2004). Recently, we reported several novel long bipyridyl-type linking ligands including ligand *L* and their coordination polymers of several transition metals (Min *et al.*, 2006; Huh *et al.*, 2008). As a continuation of our research, we decided to use ligand *L* to prepare novel platinum polynuclear or coordination network compounds. The layer diffusion (dichloromethane–benzene) of [Pt(SMe_2_)_2_Cl_2_] with an equimolar amount *L* with dichloromethane and benzene as solvents gave an unexpected dinuclear [Pt_2_L(SMe_2_)_2_Cl_4_] compound. Moreover, the reaction involving 2 equiv of *L* also gave the same product. The molecular structure of the title compound is shown Fig. 1. The complex molecule is centrosymmetric with the Pt^II^ ion exhibiting a slightly distorted square-planar coordination geometry. Each Pt atom is coordinated by two *trans* chloro ligands, one sulfur atom of SMe_2_, and pyridine N atom of ligand *L*. The PtCl_2_SN core is essentially planar with the highest displacement of 0.012 (2) Å for the Pt atom. Within the molecule, the distance between Pt atoms is 20.262 (5) Å, and the N···N separation between the terminal pyridyl rings of 16.23 (1) Å is somewhat longer than that of the free ligand (16.0 Å; Min *et al.*, 2006).

### Experimental

A dichloromethane solution (7 ml) of *L* (40 mg, 0.136 mmol) was layered onto the top of a benzene solution (7 ml) of Pt(SMe_2_)_2_Cl_2_ (50 mg, 0.130 mmol) (Hill *et al.*, 1998). Yellow crystals of [Pt_2_L(SMe_2_)_2_Cl_4_] formed in 5 days (53 mg, 0.053 mmol, 39%). The title compound is insoluble in common organic solvents. Anal. Calcd for C_26_H_28_N_4_S_2_Cl_2_Pt_2_ (Mr = 992.62): C 31.46; H 2.84; N, 5.65; S 6.46. Found: C 31.32; H 2.87; N 6.03; S 6.65. IR (KBr, ν, cm^-1^): 1620 (*s*), 1590 (*s*), 1402 (*s*), 1378 (*m*), 1308 (*s*), 1197 (*m*), 1036 (*m*), 983 (*m*), 811 (*m*), 659 (*m*).

### Refinement

All H atoms were positioned geometrically, with C—H = 0.93-96 Å and constrained to ride on their parent atoms with U_iso_(H)=1.2U_eq_(C).

### Crystal data

**Table:**

[Pt_2_Cl_4_(C_22_H_16_N_4_)(C_2_H_6_S_1_)_2_]	*Z* = 1
*M**_r_* = 992.62	*F*_000_ = 468
Triclinic, *P*1	*D*_x_ = 2.143 Mg m^−^^3^
Hall symbol: -P 1	Mo *K*α radiation λ = 0.71073 Å
*a* = 5.172 (2) Å	Cell parameters from 21 reflections
*b* = 7.2482 (11) Å	θ = 4.7–12.5º
*c* = 20.728 (3) Å	µ = 9.59 mm^−^^1^
α = 91.596 (12)º	*T* = 293 (2) K
β = 91.974 (19)º	Block, yellow
γ = 97.804 (17)º	0.44 × 0.20 × 0.10 mm
*V* = 769.0 (3) Å^3^	

### Data collection

**Table:**

Siemens P4 diffractometer	*R*_int_ = 0.042
Radiation source: sealed tube	θ_max_ = 25.1º
Monochromator: graphite	θ_min_ = 2.0º
*T* = 293(2) K	*h* = −6→0
ω scans	*k* = −8→8
Absorption correction: ψ scan(North *et al.*, 1968)	*l* = −24→24
*T*_min_ = 0.115, *T*_max_ = 0.383	3 standard reflections
3026 measured reflections	every 97 reflections
2696 independent reflections	intensity decay: none
2447 reflections with *I* > 2σ(*I*)	

### Refinement

**Table:**

Refinement on *F*^2^	Secondary atom site location: difference Fourier map
Least-squares matrix: full	Hydrogen site location: inferred from neighbouring sites
*R*[*F*^2^ > 2σ(*F*^2^)] = 0.036	H-atom parameters constrained
*wR*(*F*^2^) = 0.093	*w* = 1/[σ^2^(*F*_o_^2^) + (0.0492*P*)^2^ + 1.8521*P*] where *P* = (*F*_o_^2^ + 2*F*_c_^2^)/3
*S* = 1.05	(Δ/σ)_max_ = 0.001
2696 reflections	Δρ_max_ = 0.73 e Å^−^^3^
172 parameters	Δρ_min_ = −0.96 e Å^−^^3^
Primary atom site location: structure-invariant direct methods	Extinction correction: none

### Special details

**Table:**

Geometry. All esds (except the esd in the dihedral angle between two l.s. planes) are estimated using the full covariance matrix. The cell esds are taken into account individually in the estimation of esds in distances, angles and torsion angles; correlations between esds in cell parameters are only used when they are defined by crystal symmetry. An approximate (isotropic) treatment of cell esds is used for estimating esds involving l.s. planes.
Refinement. Refinement of *F*^2^ against ALL reflections. The weighted *R*-factor *wR* and goodness of fit *S* are based on *F*^2^, conventional *R*-factors *R* are based on *F*, with *F* set to zero for negative *F*^2^. The threshold expression of *F*^2^ > σ(*F*^2^) is used only for calculating *R*-factors(gt) *etc*. and is not relevant to the choice of reflections for refinement. *R*-factors based on *F*^2^ are statistically about twice as large as those based on *F*, and *R*- factors based on ALL data will be even larger.

### Fractional atomic coordinates and isotropic or equivalent isotropic displacement parameters (Å^2^)

**Table:**

	*x*	*y*	*z*	*U*_iso_*/*U*_eq_	
Pt1	−0.76885 (6)	0.00003 (4)	0.835419 (14)	0.05066 (13)	
Cl2	−0.8555 (5)	−0.2416 (3)	0.76008 (12)	0.0684 (5)	
Cl1	−0.6804 (6)	0.2461 (3)	0.90917 (11)	0.0783 (7)	
S1	−1.0252 (5)	−0.1418 (3)	0.91231 (11)	0.0646 (5)	
N1	−0.5382 (12)	0.1450 (8)	0.7698 (3)	0.0477 (13)	
N2	0.1135 (13)	0.4034 (9)	0.6076 (3)	0.0539 (15)	
C1	−0.3549 (16)	0.0685 (10)	0.7380 (4)	0.0536 (18)	
H1	−0.3308	−0.0536	0.7461	0.064*	
C2	−0.2008 (16)	0.1659 (11)	0.6934 (4)	0.0545 (18)	
H2	−0.0744	0.1100	0.6723	0.065*	
C3	−0.2373 (16)	0.3491 (11)	0.6804 (3)	0.0515 (17)	
C4	−0.4212 (15)	0.4285 (10)	0.7142 (4)	0.0509 (17)	
H4	−0.4454	0.5514	0.7075	0.061*	
C5	−0.5705 (15)	0.3241 (11)	0.7584 (4)	0.0505 (16)	
H5	−0.6954	0.3784	0.7807	0.061*	
C6	−0.0727 (15)	0.4623 (11)	0.6348 (3)	0.0508 (17)	
H6	−0.1095	0.5813	0.6260	0.061*	
C7	0.2847 (15)	0.5245 (11)	0.5700 (4)	0.0515 (17)	
C8	0.3580 (18)	0.7091 (12)	0.5875 (4)	0.060 (2)	
H8	0.2830	0.7612	0.6226	0.072*	
C9	0.5474 (18)	0.8204 (12)	0.5523 (4)	0.063 (2)	
H9	0.5932	0.9456	0.5641	0.076*	
C10	0.3377 (16)	0.2520 (11)	0.4981 (4)	0.0577 (19)	
H10	0.2125	0.1766	0.5201	0.069*	
C11	0.4057 (15)	0.4428 (11)	0.5180 (3)	0.0491 (16)	
C12	−1.208 (3)	−0.3570 (17)	0.8815 (7)	0.104 (4)	
H12A	−1.3382	−0.3308	0.8502	0.156*	
H12B	−1.2914	−0.4229	0.9164	0.156*	
H12C	−1.0920	−0.4321	0.8615	0.156*	
C13	−0.803 (2)	−0.2317 (17)	0.9678 (5)	0.089 (3)	
H13A	−0.6900	−0.1301	0.9888	0.134*	
H13B	−0.7003	−0.3103	0.9447	0.134*	
H13C	−0.9001	−0.3027	0.9996	0.134*	

### Atomic displacement parameters (Å^2^)

**Table:**

	*U*^11^	*U*^22^	*U*^33^	*U*^12^	*U*^13^	*U*^23^
Pt1	0.0554 (2)	0.04379 (18)	0.05348 (19)	0.00442 (12)	0.01683 (13)	0.00739 (12)
Cl2	0.0754 (14)	0.0514 (10)	0.0776 (13)	0.0024 (9)	0.0253 (11)	−0.0063 (9)
Cl1	0.1031 (18)	0.0645 (13)	0.0607 (12)	−0.0161 (12)	0.0249 (12)	−0.0036 (10)
S1	0.0712 (14)	0.0539 (11)	0.0696 (13)	0.0034 (10)	0.0301 (11)	0.0115 (9)
N1	0.043 (3)	0.048 (3)	0.052 (3)	0.003 (3)	0.008 (3)	0.006 (3)
N2	0.051 (4)	0.065 (4)	0.047 (3)	0.006 (3)	0.013 (3)	0.012 (3)
C1	0.056 (4)	0.043 (4)	0.063 (4)	0.004 (3)	0.020 (4)	0.012 (3)
C2	0.052 (4)	0.058 (4)	0.057 (4)	0.016 (3)	0.017 (3)	0.003 (3)
C3	0.058 (5)	0.057 (4)	0.039 (3)	0.006 (3)	0.012 (3)	0.009 (3)
C4	0.052 (4)	0.049 (4)	0.055 (4)	0.011 (3)	0.015 (3)	0.016 (3)
C5	0.047 (4)	0.058 (4)	0.048 (4)	0.006 (3)	0.014 (3)	0.005 (3)
C6	0.048 (4)	0.060 (4)	0.047 (4)	0.011 (3)	0.009 (3)	0.013 (3)
C7	0.052 (4)	0.059 (4)	0.047 (4)	0.012 (3)	0.016 (3)	0.017 (3)
C8	0.068 (5)	0.061 (5)	0.054 (4)	0.013 (4)	0.022 (4)	0.013 (4)
C9	0.071 (6)	0.056 (5)	0.062 (5)	0.005 (4)	0.018 (4)	0.014 (4)
C10	0.058 (5)	0.055 (4)	0.060 (4)	0.004 (4)	0.017 (4)	0.012 (4)
C11	0.051 (4)	0.055 (4)	0.043 (4)	0.010 (3)	0.011 (3)	0.016 (3)
C12	0.107 (9)	0.083 (7)	0.112 (9)	−0.034 (7)	0.030 (8)	0.018 (7)
C13	0.104 (9)	0.101 (8)	0.066 (6)	0.016 (7)	0.022 (6)	0.026 (5)

### Geometric parameters (Å, °)

**Table:**

Pt1—N1	2.057 (6)	C5—H5	0.9300
Pt1—S1	2.285 (2)	C6—H6	0.9300
Pt1—Cl2	2.301 (2)	C7—C8	1.376 (12)
Pt1—Cl1	2.303 (2)	C7—C11	1.420 (11)
S1—C13	1.795 (12)	C8—C9	1.418 (12)
S1—C12	1.799 (12)	C8—H8	0.9300
N1—C1	1.344 (10)	C9—C10^i^	1.347 (12)
N1—C5	1.357 (10)	C9—H9	0.9300
N2—C6	1.250 (10)	C10—C9^i^	1.347 (12)
N2—C7	1.427 (9)	C10—C11	1.426 (11)
C1—C2	1.386 (11)	C10—H10	0.9300
C1—H1	0.9300	C11—C11^i^	1.435 (14)
C2—C3	1.398 (11)	C12—H12A	0.9600
C2—H2	0.9300	C12—H12B	0.9600
C3—C4	1.378 (11)	C12—H12C	0.9600
C3—C6	1.484 (10)	C13—H13A	0.9600
C4—C5	1.392 (10)	C13—H13B	0.9600
C4—H4	0.9300	C13—H13C	0.9600
			
N1—Pt1—S1	175.98 (18)	N2—C6—H6	118.8
N1—Pt1—Cl2	88.55 (19)	C3—C6—H6	118.8
S1—Pt1—Cl2	95.31 (8)	C8—C7—C11	120.0 (7)
N1—Pt1—Cl1	90.40 (19)	C8—C7—N2	122.0 (7)
S1—Pt1—Cl1	85.74 (8)	C11—C7—N2	117.5 (7)
Cl2—Pt1—Cl1	178.83 (8)	C7—C8—C9	120.2 (8)
C13—S1—C12	99.7 (7)	C7—C8—H8	119.9
C13—S1—Pt1	105.2 (4)	C9—C8—H8	119.9
C12—S1—Pt1	111.4 (4)	C10^i^—C9—C8	121.2 (8)
C1—N1—C5	118.8 (6)	C10^i^—C9—H9	119.4
C1—N1—Pt1	122.1 (5)	C8—C9—H9	119.4
C5—N1—Pt1	119.1 (5)	C9^i^—C10—C11	120.8 (7)
C6—N2—C7	120.3 (7)	C9^i^—C10—H10	119.6
N1—C1—C2	122.1 (7)	C11—C10—H10	119.6
N1—C1—H1	119.0	C7—C11—C10	122.2 (7)
C2—C1—H1	119.0	C7—C11—C11^i^	119.2 (9)
C1—C2—C3	119.4 (7)	C10—C11—C11^i^	118.5 (9)
C1—C2—H2	120.3	S1—C12—H12A	109.5
C3—C2—H2	120.3	S1—C12—H12B	109.5
C4—C3—C2	118.4 (7)	H12A—C12—H12B	109.5
C4—C3—C6	119.7 (7)	S1—C12—H12C	109.5
C2—C3—C6	121.8 (7)	H12A—C12—H12C	109.5
C3—C4—C5	119.8 (7)	H12B—C12—H12C	109.5
C3—C4—H4	120.1	S1—C13—H13A	109.5
C5—C4—H4	120.1	S1—C13—H13B	109.5
N1—C5—C4	121.6 (7)	H13A—C13—H13B	109.5
N1—C5—H5	119.2	S1—C13—H13C	109.5
C4—C5—H5	119.2	H13A—C13—H13C	109.5
N2—C6—C3	122.4 (7)	H13B—C13—H13C	109.5

Symmetry codes: (i) −*x*+1, −*y*+1, −*z*+1.
